# Epidemiology of brain tumors in children aged two and under: A 10-year single-institute study

**DOI:** 10.3892/ol.2015.2943

**Published:** 2015-02-09

**Authors:** JIANZHONG YU, WE SHI, RUI ZHAO, XIAOFENG GAO, HAO LI

**Affiliations:** Department of Neurosurgery, Children’s Hospital of Fudan University, Shanghai 201102, P.R. China

**Keywords:** brain tumor, children, infant, surgical resection

## Abstract

The aim of present study was to evaluate the incidence, clinical symptoms, pathological characteristics, surgical treatment strategies and prognosis of brain tumors in children aged two and under. The current study obtained data regarding 32 consecutive infants and young children aged two years and under, who were treated for brain tumors in the Children’s Hospital of Fudan University (Shanghai, China) between 2003 and 2013. The types of tumor, clinical manifestations, location, histological features, applied treatment strategies and outcomes were retrospectively evaluated. The male to female ratio was 1.13:1, and among a total of 32 tumors, 14 (43.8%) were suptratentorial and 18 (56.3%) were infratentorial. Intracranial hypertension was the most common onset symptom, and astrocytoma was the most common tumor type (10 cases; 31.3%), followed by ependymoma (nine cases; 28.1%) and medulloblastoma (six cases; 18.8%). Surgical tumor resection was performed in 20 patients (62.5%), who experienced a mean post-operative survival time of 67.6 months. By contrast, conservative treatment with medications was administered in 12 patients (37.5%), with a mean survival time of 25.3 months. Furthermore, four patients underwent conservative therapy combined with ventriculoperitoneal shunting to relieve intracranial pressure arising from cerebrospinal fluid accumulation, resulting in a mean survival time of 10.5 months. In conclusion, the present study indicates that surgical tumor resection may improve the overall prognosis of infants and young children aged two years and under who presented with brain tumors. In addition, ventriculoperitoneal shunts may facilitate pre- and post-operative improvement in clinical symptoms by relieving intracranial pressure; however, the shunts do not appear to increase long-term survival. Furthermore, high surgical risk is an important prognostic factor in this pediatric patient population.

## Introduction

Intracranial tumors are relatively common in infants and young children; the number of cases is second only to leukemia (1) and the tumors are the most common type of solid tumor among the pediatric malignancies and have the highest mortality rates (2). Furthermore, intracranial tumors significantly affect quality of life and are one of the major causes of infant mortality (3,4). The incidence of brain tumors in infants has reportedly been gradually increasing within recent years (5).

The clinical manifestations, location and histological features of brain tumors in infants and young children less than two years old reportedly differ by age (6). Supratentorial tumors occur more frequently than infratentorial tumors in the youngest patients. Tumor type also varies by age. In the neonatal period, germ cell tumors, particularly teratoma, are the most common tumor type (7), however, in slightly older infants, astrocytoma, a neuroepithelial tumor, predominates (8,9). The majority of intracranial tumors are low-grade tumors (5), followed by ependymomas and medulloblastomas (10).

Furthermore, the clinical manifestations of intracranial tumors are different in this age group. Increased head circumference, headaches and vomiting are typical, resulting from increased intracranial pressure or obstructive hydrocephalus (11). Non-specific clinical symptoms may include irritability, drowsiness, seizures, developmental delay, difficulty swallowing and hoarseness. As the infant skull is not fully developed, its compensatory ability is greater than that of older children. Consequently, symptoms often appear relatively late (8), delaying the diagnosis and affecting the overall prognosis. Although the typical initial symptoms of intracranial hypertension, such as vomiting, are common, the inability of young patients to articulate their symptoms increases the difficulties involved in making an early and accurate diagnosis, and often contributes to misdiagnosis (12).

The rapid growth of the immature central nervous system during the first two years of life makes it acutely sensitive to the detrimental effects of radiotherapy and chemotherapy, thus, producing relatively serious side-effects in this age group compared with older children. Treatment-associated neurological effects include leukoencephalopathy, neurological and intellectual deficits, growth retardation, and endocrinological abnormalities (13). Therefore, radiography and chemotherapy are rarely prescribed in this age group, adding to the difficulty of treating brain tumors in the very young (14,15).

The development and refinement of novel neurosurgical techniques, as well as advanced diagnostic instruments has increased the importance of surgery as a treatment strategy for brain tumors (16). However, the small surgical field and the specific anesthetic requirements in children under two years old increases the difficulty of performing such surgery (17). Furthermore, the limited blood volume and relatively immature physiological functioning of infants only increases the surgical challenge (18). Despite these constraints, surgical tumor resection can significantly improve the overall prognosis of infants and children with brain tumors who are less than two years old.

The purpose of the present study was to evaluate the distinct features of pediatric neurologic neoplasms, including disease onset, clinical manifestations, histopathological features, characteristics and treatment outcomes, in infants and young children aged two years and under who presented with brain tumors.

## Patients and methods

### Patients

The present retrospective study included 32 infants aged two years and under with primary brain tumors, who were accurately diagnosed, treated and followed up between January 2003 and March 2013 in the Department of Neurosurgery at Fudan University Children’s Hospital (Shanghai, China). The protocol of the current study was approved by the Institutional Review Board of Fudan University Children’s Hospital.

### Diagnosis and classification

Diagnosis was based on a combination of three modalities: Pathological examination, clinical symptoms and diagnostic imaging. The results were obtained by performing ultrasound imaging diagnosis in combination with examination of detailed computed tomography (CT) and magnetic resonance imaging (MRI) findings. In addition, histological classification was conducted according to the 2007 World Health Organization (WHO) classification of tumors of the central nervous system (19). For the purpose of evaluation, the patients were divided into conservative treatment (n=12) and surgical intervention (n=20) groups, and data regarding disease onset, clinical manifestations, histopathological features, characteristics and treatment outcomes were compared.

### Statistical analysis

Data were analyzed using SPSS statistical software (version 18.0; SPSS, Inc., Chicago, IL, USA). Patient gender and age, tumor location and histological type, and clinical manifestations were evaluated. The associations between the treatment strategy received and the prognosis were also determined. Due to the small sample size, continuous variables are presented as the median and interquartile range (IQR). Categorical variables are presented by count and percentage. Differences between the conservative treatment and surgical intervention arms were compared using the non-parametric Mann-Whitney U test for the continuous variables and Fisher’s exact test with Yate’s correction for the categorical variables. In addition, Kaplan-Meier curves were used to calculate the overall survival and follow-up times, and the differences between the survival curves were determined by performing the log-rank test. Overall survival time was defined as the date of hospitalization to the date of mortality from the brain tumor. Patients who were alive at the time of the analysis were evaluated at the date of last contact on December 31, 2013. All statistical assessments were two-sided and P≤0.05 was considered to indicate a statistically significant difference.

## Results

### Patient demographics

The baseline characteristics of the current series of 32 pediatric patients with brain tumors are summarized in Table I. No significant differences were noted in the demographic characteristics between the two groups (P>0.05). The median age was 16.0 months (IQR, 8.3–23.3) for the conservative treatment arm and 17.5 months (IQR, 10.0–23.0) for the surgical arm. Furthermore, the median follow-up time was 18.0 months (IQR, 1.0–40.8) for the conservative treatment arm and 24.0 months (IQR, 9.3–40.5) for the surgical intervention arm. The conservative treatment arm included six (50%) males compared with 11 (55%) males in the surgical intervention arm. Five patients (41.7%) in the conservative treatment arm were infants under one year old, and seven (58.3%) were children one to two years old. By contrast, nine (45%) patients in the surgical arm were under one year old, and 11 (55%) were one to two years old. In the conservative treatment arm, six patients (50%) presented with supratentorial brain tumors and six (50%) with infratentorial brain tumors; however, in the surgical arm, eight patients (40%) presented with supratentorial brain tumors and 12 (60%) with infratentorial brain tumors. Additionally, the most common histological types in the conservative treatment group were astrocytoma, ependymoma and medulloblastoma (three cases each; 25% each); compared with astrocytoma (seven cases; 35%) and ependymoma (six cases; 30%) in the surgical arm.

### Overall survival

**Figure 1:**
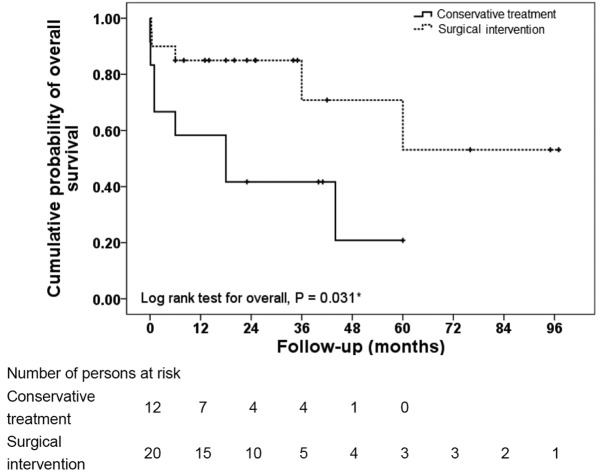
Kaplan-Meier cumulative survival curve for all subjects, stratified by treatment group (conservative treatment and surgical intervention). ^*^P<0.05 indicates a statistically significant difference.

**Figure 2:**
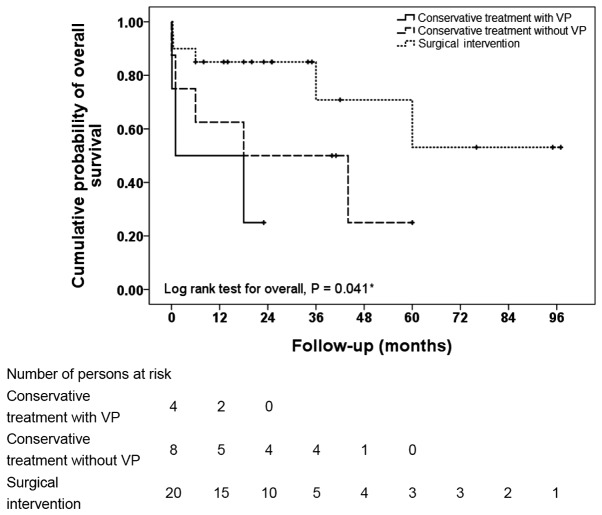
Kaplan-Meier cumulative survival curve for all subjects, stratified by treatment group (conservative treatment with VP surgery, conservative treatment without VP surgery and surgical intervention). ^*^P<0.05 indicates a statistically significant difference. VP, ventriculoperitoneal shunt.

Fig. 1 represents a comparison between the Kaplan-Meier curves of overall survival for the conservative treatment and surgical intervention arms. The survival rate was significantly higher in the surgical intervention arm (P=0.030, log-rank test). Fig. 2 indicates the Kaplan-Meier curves of overall survival for the following three treatment arms: Conservative treatment with ventriculoperitoneal shunting, conservative treatment without shunting and surgical intervention. The overall survival rate was significantly different between the three treatment groups (P=0.041, log-rank test). Patients who received surgery survived for the longest period of time, followed in order by those treated with conservative therapy alone and those treated conservatively with ventriculoperitoneal shunting.

### Diagnosis

The majority of patients were admitted to the hospital between 15 days and 4 months after disease onset, defined as the presence of symptoms based on descriptions by the patients or parents. The mean disease course was 52 days (range, 3 h to five months). The most common primary symptoms were caused by intracranial hypertension, and included headaches, nausea and vomiting (11 cases; 34.4%). Limb weakness or limited activity was observed in 10 cases (31.3%). In four cases (12.5%), the tumor was discovered during the preliminary examination. Furthermore, three patients (9.4%) experienced convulsive seizures, four (12.5%) exhibited abnormal vision and one (3.1%) exhibited precocious sexual behavior. Additionally, two patients had been transferred to Fudan University Children’s Hospital from other hospitals after being misdiagnosed with a gastrointestinal disorder.

### Treatment strategies

In total, 20 children (62.5%) underwent surgical tumor resection. Of these, 13 exhibited middle- and low-grade tumors, while seven presented with high-grade tumors; thus, the ratio between low- and high-grade tumors was ~2:1. A total of 14 patients were discharged following improvement in their symptoms or upon being considered cured, accounting for 70% of the children who underwent surgery or 43.8% of all infants and children involved in the current study. Tumors recurred in two patients (6.3%), each within the first post-operative year, and one infant (3.1%) succumbed due to post-operative sputum aspiration. Additionally, the mean survival time of the infants and children who underwent surgical tumor resection was 67.6 months [95% confidence interval (CI), 46.6–88.6 months].

Of the 12 infants and children who received conservation treatment alone, eight presented with low-grade tumors and four with high-grade tumors; thus, the ratio between low- and high-grade tumors was 2:1. The mean survival time in these patients was 25.3 months (95% CI, 11.4–39.3 months). By contrast, the mean survival time for the four patients who received conservative therapy with ventriculoperitoneal shunting was 10.5 months (95% CI, 0.6–20.5 months).

## Discussion

The clinical features, diagnosis and treatment strategies associated with brain tumors in infants and children under two years of age are unique. In the present 10-year epidemiological study of such tumors in a single institution, it was identified that a marginally greater number of males were affected than females, that more tumors were infratentorial than suptratentorial (56.2 vs. 43.8%, respectively), that intracranial hypertension was the most common onset symptom and that astrocytoma was the most common tumor type. Furthermore, prognosis differed according to the type of treatment received: Surgical tumor resection was associated with a mean post-operative survival time of 67.6 months; conservative treatment with medications and observation was associated with a mean survival time of 25.3 months; and the four patients who received conservative therapy combined with ventriculoperitoneal shunting exhibited a mean survival time of 10.5 months.

The incidence of brain tumors in children is reported to be gradually increasing (1). Although the exact reasons behind this increasing trend are unknown, we propose that it is due to a combination of factors. For example, advanced imaging techniques have improved the overall ability to diagnose brain tumors in this age group, therefore, it is possible that the actual incidence has not increased, but that tumors are being diagnosed earlier. Additionally, factors such as global environmental effects and exposure to various carcinogens may be contributing to a real increase in the incidence of brain tumors (1). However, in the current study, which included all infants and young children admitted to Fudan University Children’s Hospital in a 10-year period between 2003 and 2013, no increase occurred in the number of brain tumors in children aged two years and under. Furthermore, the types of tumor observed in the present study were generally common types; only one atypical teratoid rhabdoid tumor was identified, however, no embryonal tumors or other infant tumors that may be expected in a series of this size were diagnosed.

The occurrence of brain tumors in children who are less than two years old is associated with distinct patterns. For example, males are marginally more likely than females to develop brain tumors (6), as supported by the cases analyzed in the present study. Additionally, supratentorial tumors are reported to occur at a marginally higher rate than other types of tumors (8) Furthermore, incidence increases with increasing age, therefore, patients who are closer to two years of age exhibit a marginally higher incidence of brain tumor compared with patients who are less than one year old. In addition, patients less than one year old are reported to develop more supratentorial than infratentorial tumors. This complicates the diagnosis, as the wide supratentorial space in younger children delays the appearance of clinical symptoms compared with older children (20,21). However, contradictory results were observed in the current series, with the occurrence of a marginally higher incidence (56.3%) of infratentorial brain tumors. One possible cause for this discrepancy is that the current study only represents cases from a single center and included a relatively small number of patients. Additionally, geographical and cultural factors may have contributed to the difference.

Astrocytoma, which is a neuroepithelial tumor, is reported to be the most common histological tumor type in infants under two years old (10,22). This is compatible with the findings of the current study, which identified astrocytoma as the most frequently diagnosed tumor type, occurring in 9/32 patients (28.1%). Tumors in this age group are typically located in the supratentorial and infratentorial cerebellar hemispheres, and low-grade tumors are most prevalent (23,24). The majority of infratentorial tumors in the present series were medulloblastoma and pilocytic astrocytoma, and the number of low-grade tumors was similar to the number of high-grade tumors. Furthermore, the tumors were predominantly distributed around the midline. The majority of supratentorial tumors were located in the sellar region, and the majority of the infratentorial tumors were located in the cerebellar vermis, possibly as they are embryonal in nature (10). Similar to the results reported in a previous study (10), the three most common histological tumor types observed in the current study were astrocytoma, ependymoma and medulloblastoma.

Two cases in the current study were misdiagnosed as gastrointestinal diseases in other hospitals due to unexplained vomiting. The tumors were diagnosed only after the patients were transferred to Fudan University Children’s Hospital for additional treatment, which delayed an early diagnosis and the commencement of appropriate treatment. These two cases underscore the fact that the diagnosis is often delayed in this age group. This is partially due to infants being unable to articulate their symptoms. Parents typically notice that their child is irritable most of the time and cries more often than usual, however, they attribute the symptoms to another cause (25). Physicians may also attribute the symptoms to more common causes.

Headaches are typically a feature of functional diseases in older children, whereas in infants, headaches are often the result of organic disease (26,27). Therefore, it is important to take note of headaches in infants. In the current series, a relatively high proportion of patients (10 patients; 31.3%) were admitted to the hospital exhibiting specific neurological signs, such as epilepsy or limb dysfunction..

Ultrasound imaging is an important diagnostic modality for the early detection of intracranial tumors in this age group (28). Four patients were admitted to Fudan University Children’s Hospital after ultrasound detected intracranial lesions during initial examinations, allowing for the early initiation of treatment. Additionally, enhanced CT and MRI are important for localizing and providing a qualitative diagnosis of tumors in this age group (29). The pre-operative images obtained using these techniques correlated well with the pathological findings for those infants who underwent surgical tumor resection. Thus, ultrasound imaging combined with the use of enhanced CT and MRI may facilitate a correct diagnosis of the intracranial location of the majority of common brain tumors, greatly improving the accuracy of the diagnosis (30,31).

Surgery is considered to be a direct and effective means of treating brain tumors in infants and young children, and surgeons advise that tumors should be removed as completely as possible, as the extent of resection correlates closely with overall prognosis (32,33). In a previous study, increasing the extent of resection was associated with improved survival, regardless of age, degree of disability or WHO grade, in children with malignant astrocytomas (32). However, the majority of tumors in infants grow near the midline or occur in important functional areas of the brain, therefore, it is challenging to avoid damaging these structures during surgery. Intraoperative nerve navigation facilitates the definition of the extent of tumor resection and protects important neural structures when resecting tumors maximally (34), which is important in improving the post-operative prognosis and quality of life (35). Thus, Fudan University Children’s Hospital have recently commenced the use of intraoperative nerve navigation for brain tumor resections, with the aim of maximizing patient outcomes.

The 20 patients who underwent surgical tumor resection in the current series exhibited relatively satisfactory prognoses. A total of 14 patients were discharged after their symptoms improved or when they were considered cured, and the mean survival time of 67.6 months was a significant improvement on the 25.3-month mean survival time of the patients who received conservative treatment (P=0.030). However, surgery may not be an option for critically ill patients who would not survive the procedure or who would not derive any benefits from surgical tumor excision. Therefore, high surgical risk is a prognostic factor for pediatric patients. Infants who are not candidates for surgery are typically prescribed medication, which may be combined with ventriculoperitoneal shunting to relieve clinical symptoms (36).

In the present study, single ventriculoperitoneal shunting was performed in four infants with severe obstructive hydrocephalus secondary to tumor compression who were not candidates for surgery. Although the shunts relieved the high intracranial pressure and clinical symptoms caused by the obstructed cerebrospinal fluid, the mean survival time of 10.5 months was significantly less than the mean survival time of the infants who were treated using surgery or conservative therapy alone (P=0.041). One possible explanation for this significant difference is that these infants were more critically ill than those who were treated with conservative therapy alone. Two of the four patients presented with high-grade tumors and two with low-grade tumors; this ratio of high-grade tumors in the ventriculoperitoneal shunting treatment group is marginally higher compared with the non-ventriculoperitoneal shunting and conservative therapy group. Additionally, it is possible that single ventriculoperitoneal shunting may accelerate tumor spread in the peritoneal cavity or otherwise aggravate the disease (37).

In the present study, two patients experienced tumor recurrence within the first post-operative year, at an incidence rate of 10% of the total surgical cohort. One patient presented with a low-grade tumor and the other with a high-grade tumor. According to the WHO classification system, the ratio between low- and high-grade tumors is similar in infants who undergo surgical treatment and those who receive conservative treatment (19).

In the present study, post-operative recurrence and mortality predominantly occurred in patients whose tumors were located near vital centers, such as the brain stem or ventricle. These areas are of relatively high surgical risk, and it is difficult to completely remove the affected tissue (38). One such patient in the present study succumbed post-operatively due to aspirating sputum.

The present study was a retrospective chart review and, thus, had certain inherent limitations, which included limited patient histories and a small sample size. In addition, the current study included a relatively limited series of 32 infants who were treated for brain tumors at Fudan University Children’s Hospital and were available for follow-up. Patients lost to follow-up were excluded, however, a comparison of the histopathological diagnoses of the two treatment groups indicated no significant difference in the tumor classification or grade. Therefore, standard deviation could be controlled for when the statistical analysis was conducted.

Pathological examination was not possible for those infants who had received conservative treatment. However, among all 32 pediatric cases of common brain tumors, ultrasound imaging diagnoses were combined with the details of enhanced CT and MRI examination, allowing the observation of relatively unique clinical manifestations of brain tumors and positioning signs in the infants and young children, which ensured the greatest degree of diagnostic accuracy.. However, errors in diagnosis are inevitable and may affect the results. Despite possible discrepancies, the ratio between the low- and high-grade tumors in the surgical cohort was similar to the ratio in the patients who received conservative therapy, demonstrating, to an extent, the reliability of the results of the present study.

In conclusion, surgical tumor resection remains a direct and effective method for treating infant brain tumors. The current study indicates that surgical tumor resection may improve the overall prognosis of infants aged two years and under who presented with brain tumors. Ventriculoperitoneal shunts may pre- and post-operatively facilitate the improvement of clinical symptoms by relieving intracranial pressure from accumulated cerebrospinal fluid; however, they do not increase long-term survival. In addition, high surgical risk is a prognostic factor in this pediatric patient population.

## Figures and Tables

**Table I:** Patient demographic characteristics at baseline.

Characteristic	Conservative treatment group (n=12)	Surgical intervention group (n=20)	Overall patients (n=32)	P-value
Age, months^a^	16.0 (8.3–23.3)	17.5 (10.0–23.0)	16.5 (9.3–23.0)	0.744
0–12^b^	5 (41.7)	9 (45.0)	14 (43.8)	0.854
13–24^b^	7 (58.3)	11 (55.0)	18 (56.3)	
Follow-up period, months^a^	18.0 (1.0–40.8)	24.0 (9.3–40.5)	21.5 (6.0–40.8)	0.326
Male gender^b^	6 (50.0)	11 (55.0)	17 (53.1)	0.784
Location^b^				0.370^c^
Supratentorial	6 (50.0)	8 (40.0)	14 (43.8)	
Saddle zone	3 (25.0)	0 (0.0)	3 (9.4)	
Hemisphere	3 (25.0)	7 (35.0)	10 (31.3)	
Lateral ventricles	0 (0.0)	1 (5.0)	1 (3.1)	
Infratentorial	6 (50.0)	12 (60.0)	18 (56.3)	
Cerebellar vermis	2 (16.7)	5 (25.0)	7 (21.9)	
Cerebellar hemisphere	2 (16.7)	3 (15.0)	5 (15.6)	
Fourth ventricle	1 (8.3)	1 (5.0)	2 (6.3)	
Posterior fossa	1 (8.3)	3 (15.0)	4 (12.5)	
Histological type^b^				0.650
Astrocytoma	3 (25.0)	7 (35.0)	10 (31.3)	
Ependymoma	3 (25.0)	6 (30.0)	9 (28.1)	
Medulloblastoma	3 (25.0)	3 (15.0)	6 (18.8)	
Craniopharyngioma	1 (8.3)	0 (0.0)	1 (3.1)	
Hemangioma	1 (8.3)	1 (5.0)	2 (6.3)	
Ganglion nerve glioma	0 (0.0)	1 (5.0)	1 (3.1)	
Teratoma	1 (8.3)	0 (0.0)	1 (3.1)	
Atypical teratoma/rhabdoid tumor	0 (0.0)	1 (5.0)	1 (3.1)	
Rhabdomyosarcoma	0 (0.0)	1 (5.0)	1 (3.1)	

Data are presented as the ^a^median and interquartile range with P-values are based on the Mann-Whitney U test; or as n (%) with P-value based on ^b^Fisher’s exact test. ^c^Comparison between the supratentorial and infratentorial groups.
